# Photodynamic therapy for oral mucosal lesions: photosensitizer evolution, pharmacological mechanisms, and clinical translation

**DOI:** 10.3389/fphar.2026.1879847

**Published:** 2026-07-10

**Authors:** Guangmiao Hu, Lin Wang, Lin Fan, Jiang Sun, Dawei He

**Affiliations:** 1 School of Stomatology, Dalian Medical University, Dalian, China; 2 Department of Periodontics and Oral Mucosa Disease, Stomatological Hospital of Dalian University (Dalian Stomatological Hospital), Dalian, China

**Keywords:** drug delivery, oral mucosal lesions, photodynamic therapy, photosensitizer, reactive oxygen species

## Abstract

Oral mucosal lesions (OMLs) comprise a diverse group of diseases affecting the oral mucosal tissues. Traditional treatments such as medication, surgery, laser therapy, and cryotherapy often produce significant side effects, including high recurrence rates due to drug resistance-associated recurrence and considerable tissue trauma, leading to unsatisfactory clinical outcomes. Photodynamic therapy (PDT), which uses photosensitizers, light at specific wavelengths, and oxygen to generate cytotoxic effects that induce cell apoptosis and necrosis, cause immunogenic cell death and microcirculation damage, and trigger local immune responses, has emerged as a promising minimally invasive alternative. PDT offers advantages including strong selectivity, minimal invasiveness, high targeting capability, and repeatable administration. However, clinical translation remains constrained by pharmacological barriers including restricted tissue penetration depth of therapeutic light, absence of standardized treatment protocols, and insufficient long-term efficacy data. This comprehensive review systematically examines the evolutionary development of photosensitizer generations, explores synergistic combination strategies integrating PDT with complementary therapeutic modalities, and critically evaluates current clinical applications across common OMLs types. By synthesizing current evidence and identifying knowledge gaps regarding drug-target interactions, this review aims to provide a robust foundation for advancing therapeutic agent development and guiding future research directions in PDT-based management of OMLs.

## 1 Introduction

The oral mucosa covers the inner wall of the oral cavity, forming an important anatomical barrier structure that maintains the stability of the oral environment and resists mechanical damage. A damaged oral mucosa has weakened barrier function, which makes it easy for pathogenic microorganisms to invade and causes an imbalance in local immune defense, thereby facilitating the development of oral mucosal lesions (OMLs) (Lin et al., 2021). OMLs encompass infectious conditions (e.g., herpes simplex stomatitis and oral candidiasis), ulcerative disorders (e.g., cancer therapy-related oral mucositis), and keratotic/white patch disorders (e.g., oral leukoplakia and oral lichen planus). The cause of OMLs is complex and often delayed. Some OMLs also carry the risk of malignancy, which seriously affects the chewing and swallowing function and the quality of life of patients.

The traditional methods used for managing OMLs mainly include drug treatment, surgical treatment, laser treatment, and cryotherapy; however, these methods have certain limitations that directly motivate the search for a modality capable of selective, low-trauma ablation. Prolonged use of corticosteroids may lead to mucosal atrophy and secondary fungal infection; moreover, their withdrawal is often associated with high relapse rates, pointing to the need for a treatment that achieves lasting remission without reliance on chronic immunosuppression. Although surgical excision can eradicate focal disease, it may result in soft-tissue defects and functional impairment, underscoring the value of a tissue-sparing approach that preserves oral structure and function. The applicability of laser treatment and cryotherapy to lesions is limited; they cannot treat a wide range of lesions and cannot infiltrate deep tissue. These treatment methods are accompanied by pain, edema, and other adverse reactions after the operation. Taken together, these shortcomings converge on a clear set of design requirements for an ideal therapy: broad lesion applicability, sufficient tissue penetration, minimal collateral damage, and low post-procedural morbidity.Therefore, considering that the above existing treatments have limitations, photodynamic therapy (PDT) has emerged as a promising candidate that directly meets these criteria—its mechanism of selective photosensitizer activation enables targeted lesion destruction with deep tissue reach, sparing of surrounding normal structures, and a markedly milder side-effect profile compared to conventional modalities.

**FIGURE 1:**
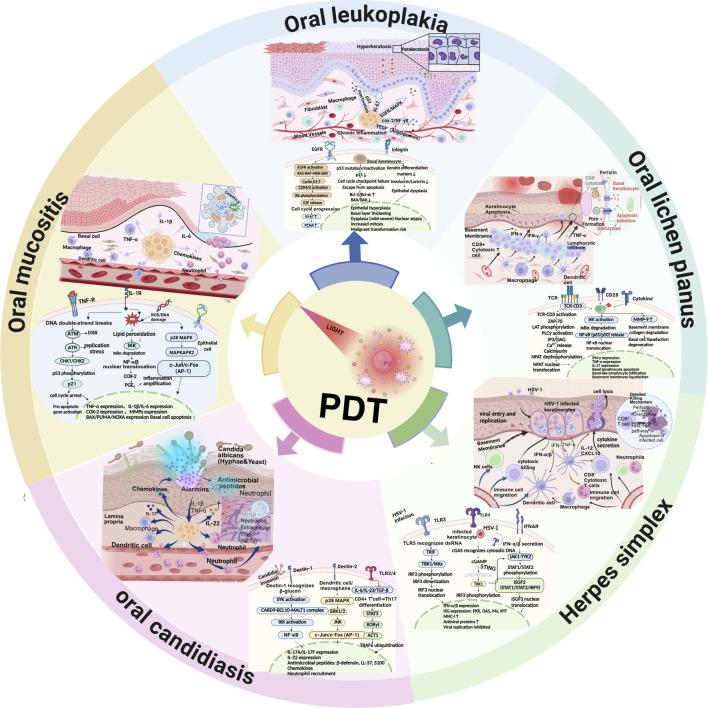
Schematic illustration depicting the utilization of photodynamic PDT in the management of oral mucosal diseases. The figure demonstrates the core mechanism involving photosensitizers and light activation to induce therapeutic effects via distinct molecular pathways specific to each pathological condition. Created with BioRender (www.biorender.com); reprinted/adapted under the BioRender Academic License.

PDT has emerged as a promising therapeutic option for OMLs. Its applications have expanded across oral leukoplakia, oral lichen planus, oral candidiasis, and related conditions, offering new avenues for clinical management (Figure 1). Simultaneously, advancements in PDT have refined its components, particularly through structural optimization of photosensitizers (PSs) and the development of novel drug delivery systems (Badran et al., 2023; Nivedita et al., 2025). However, the application of PDT in OMLs is challenged by the lack of standardized treatment protocols, a fragmented evidence base, and the absence of a systematic framework for evaluating efficacy. In this review, we synthesized recent progress in the application of PDT for OMLs and provided information on further optimization of its clinical use.

## 2 Methods

This review employed a narrative literature review approach. The literature search was conducted up to April 2026 across PubMed, Embase, Web of Science, and Scopus. The search strategy combined MeSH terms and free-text keywords including photodynamic therapy, photosensitizers, microneedling, laser therapy, immunity, oral mucosal lesions, oral leukoplakia, oral lichen planus, oral potentially malignant disorders, oral squamous cell carcinoma, oral candidiasis, oral mucositis, and related terms. Inclusion criteria were English-language original studies (human or animal), systematic reviews, meta-analyses, and high-quality narrative reviews. Studies published before 2000 or with incomplete reporting were excluded. Two authors independently screened titles, abstracts, and full texts; disagreements were resolved by consensus. As a narrative review, this search was structured but did not follow systematic review methodology.

## 3 Photodynamic therapy

PDT is a minimally invasive and targeted treatment method that selectively destroys lesioned tissues through photochemical reactions. When selectively accumulated PSs in the lesion tissue are activated by light at a specific wavelength, they transfer energy to oxygen molecules, thus producing highly cytotoxic reactive oxygen species (ROS) and other phototoxic products, which can induce cell apoptosis, necrosis, immunogenic cell death (ICD), microvascular injury, and local immune responses (Chilakamarthi and Giribabu, 2017; Kwiatkowski et al., 2018; Correia et al., 2021). PDT has multiple advantages, such as high selectivity, minimal invasiveness, targeted accuracy, and repeatability. It is widely used in the treatment of malignant tumors and precancerous lesions such as skin cancer, oral tumors, head and neck tumors, and various superficial malignant tumors (Rigual et al., 2013; Kolarikova et al., 2023; Desai et al., 2025). The application of PDT in the treatment of infectious diseases is called antibacterial photodynamic therapy (aPDT), which eliminates pathogenic microorganisms through a ROS-mediated mechanism independent of the specific target of traditional antibiotics, thus minimizing the chances of developing drug resistance. Additionally, aPDT has been widely used in the fields of oral medicine and dermatology to treat bacterial, fungal, and viral infections.

### 3.1 Photosensitizers

PSs are the core of the therapeutic effect of PDT. They selectively accumulate in the lesion tissue and trigger subsequent photochemical reactions when exposed to light irradiation of appropriate wavelengths. The molecular structure of PSs determines its optimal excitation wavelength, tissue penetration depth, and the yield of ROS (Simões et al., 2020; Dhanya et al., 2023). Consequently, next-generation PSs with optimized characteristics need to be developed to improve clinical efficacy (Table 1).

**TABLE 1:** Representative photosensitizers for photodynamic therapy.

Generation	Type	Name	Excitation wavelength	Key characteristics	References
First Generation	Porphyrin derivatives	Hematoporphyrin derivatives (HpD)	∼630 nm	Complex mixture; low tumor selectivity; slow photosensitivity resolution; cannot reach deep tissue	Dougherty (1974), Abrahamse and Hamblin (2016)
Second Generation	5-ALA derivatives	5-Aminolevulinic acid (5-ALA)	635 nm (PpIX)	Endogenous precursor converted to PpIX; selective accumulation in diseased cells; effective for superficial malignancies and OPMDs	Tetard et al. (2014), Kou et al., 2017; Lim (2018), Railkar and Agarwal (2018), Lim et al. (2022), Ou et al. (2022), Bordoloi et al. (2024), Chen et al. (2025)
	Phenothiazines	Methylene Blue (MB)	∼665 nm	Water-soluble cationic dye; low toxicity; minimal side effects; low cost; used in aPDT for oral infections and multidrug-resistant bacteria	Sridharan and Shankar (2012), Méndez et al. (2018), Minagawa et al. (2023), Cardozo et al. (2024), Ferreira et al. (2024), Jervøe-Storm et al. (2024), Petrilli et al. (2025), Wu et al. (2025)
		Toluidine Blue (TB)	∼630 nm	High affinity for nucleic acids; targets proliferative cells; used for diagnosis and treatment of OPMDs	Sridharan and Shankar (2012), Afkhami et al. (2020)
	Chlorophyll derivatives	Chlorin e6 (Ce6)	∼660 nm	High ROS quantum yield; excellent biocompatibility; low dark toxicity; rapid clearance; used for head/neck cancers and oral lesions	Pietruska et al. (2014), Ding et al. (2015), Zago et al. (2020), Cai et al. (2021), Pucci et al. (2021), Kim and Chang (2023), Liu et al. (2025), Wang et al. (2025)
	Phthalocyanines	Zinc phthalocyanine (ZnPc)	670–700 nm (NIR)	Near-infrared absorption; potential for deep tissue penetration; high triplet state quantum yield; poor water solubility	Roguin et al. (2019), Li et al. (2023)
		Aluminum phthalocyanine (AlPc)	670–700 nm (NIR)	Efficient singlet oxygen generation; long triplet lifetime; used in skin cancer, melanoma, and oral cancer treatment	Kim et al. (2021), Al-Kheraif et al. (2022), Li et al. (2023), Huang et al. (2026)
Third Generation	Liposomal formulations	MB-loaded liposomes	∼665 nm	Enhanced encapsulation efficiency (up to 83.57%); improved tumor targeting; strong bactericidal effects on oral biofilms	Malam et al. (2009), Boccalini et al. (2017), Xia et al. (2026)
		AlClPc-loaded liposomes	670–700 nm	Threefold increase in ROS production; enhanced PDT efficacy in breast cancer	Nogueira et al. (2024)
		Ce6-loaded liposomes	∼660 nm	Improved water solubility and bioavailability; enhanced antitumor immune responses by inducing apoptosis	Ma et al. (2025)
	Micellar formulations	MB-loaded micelles	∼665 nm	Enhanced stability; prolonged circulation time; effective against oral biofilms via EPR effect	Wakaskar (2018), Soares et al. (2023)
		Ce6-fluorinated micelles	∼660 nm	Overcome hypoxic microenvironment; enhanced ROS generation in hypoxic tumors	Wang et al. (2018)
​	Solid lipid nanoparticles	MPPa-loaded SLNs	∼665 nm	Improved water solubility; controlled release; enhanced anticancer effects	Graván et al. (2023), Mehrdadi (2023), Yeo et al. (2023)
		Curcumin-loaded SLNs	∼430 nm	Enhanced hydrolytic stability; improved light-induced ROS formation	Jiang et al. (2017)
	Nanostructured lipid carriers	Ang-Ce6-NLCs	∼660 nm	Active targeting via angiopep-2; increased drug loading; enhanced glioblastoma targeting; minimized damage to healthy tissues	Mehnert and Mäder (2001), Liu et al. (2011), Pucci et al. (2025)
		ClAlPc-loaded NLCs	670–700 nm	Superior skin permeability; enhanced phototoxicity against lung cancer and melanoma cells	Almeida et al. (2018)
	Metallic nanomaterials	5-ALA-loaded gold nanoparticles (AuNPs)	635 nm (PpIX)	Surface plasmon resonance enhancement; improved delivery efficiency and stability; strong tumor proliferation inhibition; enhanced diagnostic accuracy; no systemic toxicity	Khlebtsov et al. (2013), Younis et al. (2021), George et al. (2022), Soomro et al. (2024)

Ideal PSs should have the following characteristics: selective accumulation in lesions with high purity and stable composition. They should have favorable photophysical/photochemical properties, including strong absorption within 600–800 nm with high molar extinction and efficient ROS generation. Moreover, they should be safe for biological application, including minimal dark toxicity and quick removal from the body to reduce the risk of long-term phototoxicity (Correia et al., 2021).

#### 3.1.1 First-generation PSs

First-generation PSs, typically represented by hematoporphyrin derivatives (HpD) and their semi-synthetic product Photofrin®, have promoted the clinical application of modern PDT and facilitated further research and development in this field (Dougherty, 1974; Abrahamse and Hamblin, 2016). However, these agents are mixtures of porphyrins with complex compositions that have low tumor selectivity, slow resolution of cutaneous photosensitivity, and cannot reach deep tissue. These limitations have greatly promoted the advancement in the research and development of second-generation PSs.

#### 3.1.2 Second-generation PSs

To overcome the defects of the first-generation PSs, researchers have developed second-generation PSs with a well-defined chemical structure and high purity. The key advantages of these PSs include absorption maxima shifted to 650–800 nm, which improves tissue penetration, greater lesion selectivity, faster systemic clearance with a shorter duration of photosensitivity, and higher singlet oxygen yield. Representative second-generation PSs include 5-aminolevulinic acid (5-ALA) (Tetard et al., 2014; Kou et al., 2017), phenothiazines (Sridharan and Shankar, 2012; Cardozo et al., 2024), chlorophyll derivatives, and phthalocyanines.

##### 3.1.2.1 5-Aminolevulinic acid and its derivatives

The compound 5-ALA is not a PS but an endogenous precursor. After exogenous administration, proliferative lesional cells preferentially take up 5-ALA and convert it via the heme biosynthetic pathway to protoporphyrin IX (PpIX), a highly photoactive species. Endogenously synthesized PpIX accumulates selectively in diseased cells, imparting strong targeting ability and therapeutic efficacy (Tetard et al., 2014; Kou et al., 2017; Bordoloi et al., 2024). Additionally, 5-ALA-mediated PDT is widely used for superficial malignancies such as skin, bladder, and esophageal cancers, and can effectively treat human papillomavirus (HPV)-related lesions (Lim, 2018; Railkar and Agarwal, 2018; Lim et al., 2022; Chen et al., 2025). In oral medicine, 5-ALA-PDT is an important option for treating oral leukoplakia (OLK) and oral lichen planus (OLP) in oral potentially malignant disorders (OPMDs), with better long-term outcomes compared to conventional therapeutic techniques (Ou et al., 2022; Han et al., 2025a).

##### 3.1.2.2 Phenothiazines

Phenothiazines are commonly used cationic dyes, with representative agents including methylene blue (MB) and toluidine blue (TB). MB is a water-soluble cationic phenothiazinium salt compound with a maximum absorption peak at approximately 665 nm. MB has attracted widespread attention in the field of PDT owing to its low toxicity, minimal side effects, and low cost. TB is a phenothiazine derivative that serves as a basophilic metachromatic dye and has high affinity for nucleic acids. It can specifically bind to acidic components in cells, serving as a nuclear marker for tracing regions of active cell proliferation (Sridharan and Shankar, 2012). Its positively charged amphiphilic molecular properties facilitate its accumulation in target tissues, while its singlet oxygen quantum yield ensures favorable PDT outcomes.

Phenothiazine PSs are primarily used in aPDT for oral infectious diseases, including periodontitis, root canal infections, dental caries, and peri-implantitis (Méndez et al., 2018; Minagawa et al., 2023; Jervøe-Storm et al., 2024; Petrilli et al., 2025; Wu et al., 2025). Additionally, MB-PDT can effectively treat skin infections, wound infections, and multidrug-resistant bacterial infections (Ferreira et al., 2024). TB is commonly used for the diagnosis and treatment of OPMDs, effectively reducing lesion size.

##### 3.1.2.3 Chlorophyll

Chlorophyll derivatives are obtained from natural chlorophyll through chemical modification. Their advantages include low dark toxicity, rapid *in vivo* clearance, and a broad light absorption range. However, their application in PDT is severely limited due to low water solubility, tendency to aggregate, and instability in biological environments (Pucci et al., 2021). Among them, chlorin e6 (Ce6) and its derivatives are the most extensively studied; they not only possess high ROS quantum yields but also demonstrate excellent biocompatibility (Cai et al., 2021).

Chlorin e6 (Ce6)-mediated PDT has been applied in the treatment of head and neck squamous cell carcinoma, early-stage lung cancer, pancreatic cancer, and skin tumors (Ding et al., 2015; Kim and Chang, 2023). In the field of oral medicine, it has effectively treated OLK, carcinoma *in situ*, and early-stage oral squamous cell carcinoma (Pietruska et al., 2014; Liu et al., 2025). Additionally, Ce6 derivatives can strongly inactivate multispecies biofilms associated with periodontal disease (Zago et al., 2020; Wang et al., 2025).

##### 3.1.2.4 Phthalocyanines

Phthalocyanines (Pcs) are a class of synthetic dyes with large conjugated ring structures. They have maximum absorption peaks in the near-infrared region, and thus may be able to treat deep-seated lesions. Through axial coordination of different metal ions introduced at the center of the Pcs ring, when coordinated with diamagnetic metals such as zinc(II) or aluminum (III), Pcs exhibit excellent photosensitizing properties, including high triplet state quantum yields and long triplet state lifetimes, which allow efficient singlet oxygen generation (Roguin et al., 2019; Li et al., 2023). The main limitation of Pcs is their poor water solubility, which restricts their direct clinical application to some extent.

Phthalocyanine derivative-mediated PDT has been implemented for treating skin cancer, melanoma, and oral cancer (Kim et al., 2021). In recent years, phthalocyanine-antimicrobial peptide conjugates (Pc-AMP conjugates) have demonstrated strong antimicrobial effects and biofilm inhibition capabilities in the prevention of caries, with additional applications in tooth whitening (Huang et al., 2026). Chloro-aluminum phthalocyanine-mediated aPDT combined with root surface debridement can significantly decrease the levels of *Porphyromonas gingivalis* and *Tannerella forsythia* in stage III periodontitis patients and effectively suppress the expression of inflammatory factors such as TNF-α (Al-Kheraif et al., 2022).

#### 3.1.3 Nano-enabled third-generation PSs systems

##### 3.1.3.1 Liposomes

Liposomes are spherical vesicles composed of phospholipid bilayers (particle size: 50–450 nm). Their unique bilayer structure enables them to encapsulate hydrophobic PSs in the hydrophobic lipid bilayer and hydrophilic PSs in the hydrophilic core. Liposomes have satisfactory biocompatibility, biodegradability, and ease of functionalization, and thus represent a highly versatile PSs delivery system (Malam et al., 2009).

When MB is encapsulated in liposomes, its encapsulation efficiency can be increased from 37.50% with traditional passive loading to 83.57% with active loading methods, significantly increasing the plasma clearance rate and enhancing tumor targeting (Xia et al., 2026). In oral microbial therapy, MB-loaded liposomes exert strong bactericidal effects against *Streptococcus mutans* and *Candida albicans* biofilms (Boccalini et al., 2017). Chloro-aluminum phthalocyanine (AlClPc) encapsulated in cationic liposomes exhibits a threefold increase in ROS production compared to conventional PSs, significantly enhancing the efficacy of PDT in breast cancer treatment (Nogueira et al., 2024). Ce6 encapsulation in liposomes not only significantly improves its water solubility and bioavailability but also enhances anti-tumor immune responses by inducing apoptosis (Ma et al., 2025).

##### 3.1.3.2 Micelles

Micelles are formed by the self-assembly of amphiphilic copolymers in water (particle size: 10–200 nm). Their hydrophobic core can efficiently encapsulate PSs, while the hydrophilic shell helps prolong blood circulation time and promotes the accumulation of PSs in diseased tissues through the EPR effect, thereby increasing the efficiency of PDT (Wakaskar, 2018).

Methylene blue (MB)-loaded polymeric micelles have demonstrated excellent stability and antimicrobial effects in antimicrobial PDT of oral biofilms, exhibiting significant bactericidal activity against *S. mutans* and *C. albicans* biofilms (Soares et al., 2023). Fluorinated polymeric micelles of Ce6 (Ce6-PFOC-PEI-M) can overcome the hypoxic microenvironment in hypoxic C6 glioma cells, generating significantly higher ROS under laser irradiation compared to non-fluorinated controls (Ce6-OC-PEI-M), thereby exhibiting greater phototoxicity *in vitro* and achieving stronger tumor growth inhibition *in vivo* (Wang et al., 2018).

##### 3.1.3.3 Solid lipid nanoparticles

Solid lipid nanoparticles (SLNs) are composed of a solid lipid core (particle size: 40–1,000 nm) and have adequate physical stability (Graván et al., 2023). When SLNs are used as delivery carriers for PSs, they offer multiple advantages, including efficient encapsulation and delivery of hydrophobic PSs, greater bioavailability, promotion of effective targeted accumulation of PSs in diseased tissues, and poor water solubility that facilitates controlled release of PSs, avoiding initial burst release effects and maintaining effective therapeutic concentrations (Mehrdadi, 2023).

Methyl pyropheophorbide-a (MPPa) encapsulated in SLNs can significantly improve the water solubility issues of this chlorophyll-type PS and demonstrate enhanced anticancer effects of PDT *in vitro* (Yeo et al., 2023). Curcumin-loaded SLNs can increase the hydrolytic stability of curcumin and promote light-induced ROS formation, greatly increasing the killing efficiency of cancer cells exposed to light at 430 nm (Jiang et al., 2017).

##### 3.1.3.4 Nanostructured lipid carriers

Nanostructured lipid carriers (NLCs) are composed of a mixture of solid and liquid lipids that form an imperfect lattice structure. Compared to the structure of SLNs, the structure of NLCs can accommodate more PS molecules, increasing the drug loading capacity and effectively preventing drug leakage during storage (Liu et al., 2011). They are efficient carriers that deliver nucleic acids to regulate gene expression and enable gene therapy and personalized medicine. Additionally, they provide a multifunctional platform for nucleic acid therapies in genetic diseases and tumors (Mehnert and Mäder, 2001).

Researchers have found that Ce6-loaded targeted nanostructured lipid carriers (Ang-Ce6-NLCs) achieve active targeting of glioblastoma through angiopep-2 peptide modification, significantly reducing the viability of tumor cells and increasing apoptosis while minimizing damage to surrounding healthy tissues and maximizing therapeutic efficacy (Pucci et al., 2025). The system co-encapsulating chloro-aluminum phthalocyanine (ClAlPc) and oleic acid in NLCs showed greater skin permeability, biocompatibility, and anti-tumor effects compared to traditional SLNs, exhibiting stronger phototoxicity against A549 lung cancer cells and B16-F10 melanoma cells (Almeida et al., 2018).

##### 3.1.3.5 Metallic nanomaterials

Metallic nanomaterials are an important class of PS delivery carriers used in PDT. Unlike silica-based carriers, PSs are generally modified on the surface of metallic nanoparticles through covalent or non-covalent methods, and their large specific surface area provides favorable conditions for high-density loading (Khlebtsov et al., 2013). Metallic nanomaterials are mainly classified into four categories, which include (1) metallic nanoparticles (pure metal nanoparticles), (2) metal oxide nanoparticles, (3) doped metal/metal oxide nanomaterials, and (4) metal sulfides and metal-organic framework nanomaterials.

Metallic nanoparticles that act as PS carriers have the following advantages: first, they can serve as efficient energy converters, effectively increasing the light absorption efficiency of PSs and enhancing singlet oxygen quantum yields (Younis et al., 2021). Through precise synthesis and surface modification, metallic nanoparticles can specifically bind to antibodies, drugs, and targeting ligands (George et al., 2022). These properties make plasmonic metallic nanoparticles an ideal platform for optimizing the performance of traditional PSs.

Gold nanoparticles can use their surface plasmon resonance effect to enhance local electromagnetic fields under light irradiation, thereby significantly improving the efficiency of generating singlet oxygen. AuNPs loaded with 5-ALA have significant advantages in the treatment of oral squamous cell carcinoma. They not only enhance the efficiency of delivering PS and the stability of PS but also strongly inhibit tumor proliferation and improve the diagnostic accuracy, with no systemic toxicity (Soomro et al., 2024).

#### 3.1.4 Emerging PSs

Most PSs when packed together in physiological media, lose fluorescence and ROS output, this a problem called aggregation-caused quenching (ACQ). AIE compounds are bright. For OMLs, this relevant because both topical and systemic delivery end up concentrating the PSs inside the lesion, making it hard to avoid molecular crowding. The newer AIE PSs further push this: type I ROS pathways that work under low oxygen, emission in the NIR-II window, and motifs that target multiple organelles at the same time. A few have been made to oral cancer xenografts, and have shown decent tumor-to-normal selectivity (Wu et al., 2021; Yang et al., 2025). Adding lesions-microenvironment triggers, like pH, redox state, or tumor-associated enzymes, to switch PSs from silent to active only on site has been shown (Yu et al., 2024). The second direction is naturally formed by PSs, mainly chlorophyll derivatives from algae and plants (Attaoui et al., 2025). Microalgal potassium chlorophyllin has a photodynamic potency similar to synthetic agents, and remains bioactive without light (Rajaram et al., 2024). Spirulina platensis has been tested against head and neck squamous cell carcinoma lines, and it has a low toxicity to normal cells (Saberi et al., 2022). Other entries include diatom-derived chlorophyll carriers, pH-sensitive chlorophyll–peptide conjugates, and chlorophyll loaded into mesoporous silica to enhance its photodynamic yield (Adnane et al., 2024; Nagatani et al., 2025). None of these have been tested clinically in the oral cavity yet.

### 3.2 Light

Light sources are an important component of PDT systems. Its wavelength, energy density, and irradiation mode directly affect the efficiency of activation and therapeutic effect of PS. The selection of the light source needs to be accurately matched with the absorption characteristics of PS, and sufficient tissue penetration depth must be ensured (Wilson and Patterson, 2008). Based on the type of light source, the PDT light source is mainly divided into lasers and lamps.

#### 3.2.1 Lasers

Lasers are the most widely used source of light in PDT. Their main advantages include good monochrome, high energy density, controllable penetration depth, and accurate shape of the light spot. The monochromaticity of lasers ensures that light energy is efficiently absorbed by PS, and high energy density and dimmable spots are especially suitable for the precise irradiation of small areas or deep lesions.

Commonly used sources of clinical lasers include semiconductor lasers, gas lasers, and dye lasers. Semiconductor lasers are the most commonly used PDT light source. The wavelength can cover 630–690 nm, which closely matches the absorption peaks of most second-generation PSs. They are small, highly stable, and inexpensive. Gas lasers such as helium-neon lasers and argon ion lasers have extremely high spectral purity, but they require large equipment, have a high maintenance cost, and have limited clinical applications. The dye laser takes organic dye as the gain medium and can output 600–650 nm tunable lasers. By adjusting the composition of the dye, the wavelength can be adjusted to match the excitation needs of different PSs. Solid-state lasers (such as Nd:YAG lasers) can achieve precise matching with the absorption peaks of PSs and produce highly uniform beams, helping shorten the treatment time (Brancaleon and Moseley, 2002).

#### 3.2.2 Lamps

Lamp sources are commonly used in PDT for treating lesions that are superficial or have a large surface area. These sources mainly include light-emitting diodes (LEDs), halogen lamps, and xenon lamps. LEDs can serve as semiconductor devices based on spontaneous radiative emission from electron-hole recombination and provide multiple wavelength options, such as 630–660 nm. The spectral bandwidth of LEDs is approximately 5% of the center wavelength; although they are inferior to laser monochromaticity, they demonstrate good wavelength compatibility with commonly used PSs and have several advantages, such as low energy consumption, long lifespan, small size, and excellent portability. They can be designed as array-type irradiation heads to achieve uniform illumination over a large area (Brancaleon and Moseley, 2002). Halogen lamps and xenon lamps are broad-spectrum light sources that emit continuous broadband spectra at approximately 300–1,200 nm; these lamps require optical filters to select specific wavelengths and remove interference from ultraviolet and infrared bands. Although their spectral purity is lower than that of LEDs, their large irradiation area makes them suitable for the simultaneous treatment of multiple oral sites.

#### 3.2.3 Daylight

Daylight is a special form of PDT where natural sunlight is used as the light source. It offers the advantages of having no equipment cost, a continuous spectrum, and being well-tolerated by patients. The underlying principle involves dependence on the visible light band in daylight for the activation of various PSs, especially 5-ALA and its derivatives. Unlike high-energy-density monochromatic light PDT, daylight PDT uses outdoor low-energy continuous irradiation, resulting in a gentler process of activating PSs with lower ROS generation rates, significantly reducing pain and inflammatory responses in patients (Wong and Morton, 2025).

### 3.3 Oxygen

Oxygen serves as an important substrate in PDT photochemical reactions. Both Type I reactions, which generate free radicals, and Type II reactions, which produce singlet oxygen, require the participation of oxygen molecules. Under anoxic or severely hypoxic conditions, even when PSs are photoexcited, they cannot generate sufficient ROS, and the cytotoxic effects of PDT are greatly reduced or even completely lost. Therefore, adequate oxygen supply is a prerequisite for ensuring high efficacy of PDT (Angjelova et al., 2023).

### 3.4 PDT molecular mechanism

**FIGURE 2:**
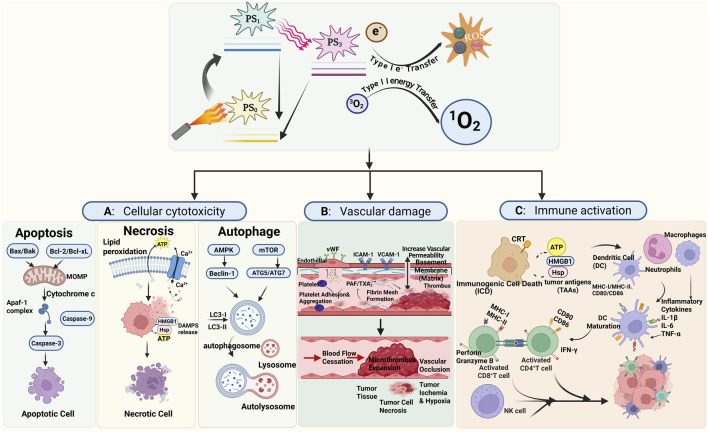
Schematic illustration of photosensitizer-mediated PDT mecha-nisms. Following light activation at an appropriate wavelength, the photosensitizer generates ROS, including singlet oxygen (^1^O_2_), through Type I and Type II photochemical reactions. These ROS exert therapeutic effects through three principal pathways: **(A)** cellular cytotoxicity via apoptosis, necrosis, and autophagy; **(B)** vascular damage leading to endothelial injury, thrombosis, and tissue ischemia; and **(C)** immune activation through immunogenic cell death (ICD)-mediated release of damage-associated molecular patterns (DAMPs) and subsequent adaptive immune response. Created with BioRender (www.biorender.com); reprinted/adapted under the BioRender Academic License.

The mechanisms underlying PDT can be elucidated from two interrelated perspectives: the mechanism underlying the photochemical reaction and the mechanism underlying the biological effect. The former focuses on the physicochemical process by which the PS, after being excited by a light source of a specific wavelength, generates ROS through Type I and Type II photochemical reaction pathways; the latter focuses on how ROS exert comprehensive therapeutic effects on pathological tissues through multilevel biological pathways, including cellular cytotoxicity, vascular damage, and immune activation (Figure 2).

#### 3.4.1 Photochemical reaction mechanisms

PDT utilizes molecular oxygen through two pathways (Type I and Type II reactions), generating different types of ROS at lesion sites. After PSs accumulate at the lesion site, ground-state PS (^1^PS) is photoactivated and transforms into an extremely short-lived excited singlet state (^1^PS*). As this state is unstable, it is converted to a more stable excited triplet state (^3^PS*) through intersystem crossing, which can transfer energy from the environment to biomolecules. Type I mechanism: triplet state PS (^3^PS*) reacts with substrates in diseased tissue, transferring hydrogen or electrons to form free radical intermediates (radicals and anion radicals) between the PS and substrate. These intermediates react with oxygen molecules, generating various ROS, including superoxide anion (O_2_^−^), hydrogen peroxide (H_2_O_2_), and hydroxyl radical (·OH). Type I reactions are more pronounced in hypoxic microenvironments or with lipophilic PSs. Type II mechanism: ^3^PS* directly transfers energy to molecular oxygen, generating highly reactive ^1^O_2_ (Kwiatkowski et al., 2018).

Type I and Type II reactions coexist and compete with each other, with their occurrence regulated by multiple factors, including the type of PS, local oxygen concentration, substrate concentration, and light irradiation parameters (Dolmans et al., 2003; Baptista et al., 2017). The primary cytotoxic agent is ^1^O_2_; therefore, Type II reactions typically dominate. However, as the duration of light treatment increases, the gradual consumption of oxygen exacerbates hypoxia in diseased tissues, causing Type I reactions to gradually become dominant and decreasing the efficacy of PDT.

#### 3.4.2 Mechanisms of biological effects

ROS generated through photochemical reactions exert therapeutic effects on pathological tissues via multilevel biological pathways. At the cellular level, ROS-induced oxidative stress can trigger three principal modes of cell death. Apoptosis is a type of programmed cell death characterized by orderly cell shrinkage, chromatin condensation, DNA fragmentation, and the formation of apoptotic bodies. At low doses, ROS preferentially initiate the mitochondria-mediated apoptotic program, manifested by upregulation of the pro-apoptotic proteins Bax and Bak, and downregulation of the anti-apoptotic proteins Bcl-2 and Bcl-xL, leading to the release of cytochrome c. This subsequently activates the apoptotic protease-activating factor-1 (Apaf-1) complex and the caspase-9/caspase-3 cascade, which results in orderly cellular dismantling (van Straten et al., 2017; Kessel and Reiners, 2020). Necrosis is a pathological form of cell death characterized by the loss of integrity of the plasma membrane, organelle swelling, and leakage of intracellular contents, which triggers an inflammatory response. At high doses, ROS can induce the peroxidation of membrane lipids, acute depletion of ATP, and calcium overload, triggering rapid rupture of the plasma membrane and the release of damage-associated molecular patterns (DAMPs), including lactate dehydrogenase (LDH) and high mobility group box 1 (HMGB1), thereby triggering necrotic cell death (Chilakamarthi and Giribabu, 2017; Kessel, 2020; Ding et al., 2025). Autophagy is a self-digestive process through which cells degrade damaged organelles and macromolecules in lysosomes to maintain cellular homeostasis. Moderate levels of oxidative stress can trigger AMP-activated protein kinase (AMPK) and inhibit the mammalian target of rapamycin (mTOR), initiating Beclin-1-mediated autophagy. This process promotes the conversion of microtubule-associated protein light chain 3-I (LC3-I) to LC3-II through the autophagy-related proteins ATG5 and ATG7, facilitating the formation of autophagosomes and subsequent fusion with lysosomes to generate autolysosomes, thereby regulating cell fate (Kessel and Reiners, 2007; Kessel, 2015; 2019). These three modes of cell death frequently coexist and interact during PDT of OMLs, and their relative proportions are determined by factors such as the dosage of PS, light irradiation parameters, and types of cells.

At the vascular level, PDT can cause microvascular endothelial damage in tumorous or dysplastic tissues. Injured endothelial cells exhibit higher expression of several markers, including von Willebrand factor (vWF), intercellular adhesion molecule-1 (ICAM-1), vascular cell adhesion molecule-1 (VCAM-1), and endothelin-1 (ET-1), accompanied by an increase in vascular permeability. Subsequently, platelets adhere and aggregate, leading to the formation of a fibrin mesh and thrombus under the action of thromboxane A2 (TXA2) and platelet-activating factor (PAF), which results in vasoconstriction and cessation of blood flow. Sustained blood stasis induces tissue hypoxia and ischemia, which in turn upregulates hypoxia-inducible factor-1α (HIF-1α) and vascular endothelial growth factor (VEGF), ultimately causing secondary necrosis of the lesional tissue (Bhuvaneswari et al., 2009; Agostinis et al., 2011; Yang et al., 2021). This vascular effect plays a significant role in the treatment of oral precancerous lesions such as OLK.

Additionally, PDT exerts immunomodulatory effects. PDT-induced photodamage to tumor cells can trigger ICD, thereby eliciting a local acute inflammatory response and activating the innate immune system. ROS-mediated cellular damage leads to the release of DAMPs, including calreticulin (CRT) translocated from the endoplasmic reticulum to the surface of the cell membrane, extracellularly released adenosine triphosphate (ATP) and HMGB1, and exposed or secreted heat shock protein 70/90 (HSP70/HSP90), as well as tumor-associated antigens (TAAs) (Castano et al., 2006; Beltrán Hernández et al., 2020).

Antigen-presenting cells, such as dendritic cells (DCs) and macrophages, recognize these “danger signals” and then rapidly recruit neutrophils, macrophages, and DCs to the site of inflammation. When activated, immune cells release large amounts of pro-inflammatory cytokines, including IL-1β, IL-6, and TNF-α, thereby promoting the maturation of DCs. Mature DCs take up and process TAAs, upregulate the expression of peptide-major histocompatibility complex class I/II molecules (peptide-MHC-I/II) and co-stimulatory molecules (CD80/CD86). Eventually, they migrate to draining lymph nodes to present antigens to naïve T cells, thus initiating adaptive immunity. The PDT-induced innate immune response lays the foundation for adaptive immunity. Activated CD8^+^ cytotoxic T lymphocytes directly kill tumor cells by releasing perforin and granzyme B, while CD4^+^ helper T cells secrete interferon-γ (IFN-γ) to enhance the immune response and activate natural killer (NK) cells. NK cells function synergistically with CD8^+^ T cells, establishing a dual cytotoxic mechanism (Donohoe et al., 2019; Beltrán Hernández et al., 2020; Nkune et al., 2021). Partially activated T cells differentiate into memory T cells, which exist in lymphatic organs and tissues for a long time, providing long-term immune surveillance. Finally, PDT elicits an adaptive immune response with antigen-specificity and immune memory function to develop systemic anti-tumor immunity, which not only removes local tumors but also prevents recurrence and metastasis. In this multi-target comprehensive action model, PDT has a unique advantage in treating OMLs.

## 4 Combined treatment strategies of PDT

Although PDT can effectively treat OMLs, monotherapy is often constrained by limited tissue penetration depth and suboptimal bioavailability, particularly in lesions that are deep or have a large surface area.​ To further improve the therapeutic effect, researchers have investigated the combined application of PDT with other treatment methods. These combined therapeutic strategies can overcome the limitations of single therapy through synergistic pharmacological effects and provide new treatment options for complex or incurable OMLs. However, these combined strategies are not without drawbacks. Each modality carries specific risks or challenges that warrant careful consideration.

### 4.1 PDT + microneedles

Microneedles (MNs) are micron-scale arrays that form channels on the surface of the mucosa. The structure of the oral mucosa limits the percutaneous absorption of PSs, and MNs serve as an effective transmucosal drug delivery system, which can significantly increase the delivery efficiency of PSs to the deep layer of the lesion. Accurate local administration significantly improves the delivery of PSs to deep tissues, and PSs do not enter the somore circulation. This helps overcome the problem of poor penetration of Ps in PDT (Sartawi et al., 2022). After the soluble microneedle loads PSs, it is applied at the lesion site. The microchannel formed when MNs dissolve and release PSs further promotes the diffusion of PSs; this can increase the concentration of PSs in the target tissue by 2–3 times. The combined application of PDT and MNs can be effective for diseases requiring deep treatment, such as keratinized OLK and refractory OLP (Wang et al., 2021; Han et al., 2025b; Sang et al., 2025). The combined strategy can improve the photodynamic treatment effect of vitiligo lesions and reduce the risk of recurrence and malignancy. Nevertheless, microneedle insertion may disrupt the mucosal barrier physically, leading to transient pain, local bleeding, and mild inflammatory reactions at the application site (Jeong et al., 2017; Nguyen and Park, 2018; Moawad et al., 2025). Moreover, the microchannels created by MNs temporarily affect the integrity of the oral mucosa, which may allow bacterial entry and increase the risk of local infection, especially in the pathogen-rich oral environment. These drawbacks, although generally self-limiting, might affect patient compliance, especially in people with low pain tolerance or those needing repeated sessions.

### 4.2 PDT + lasers

As high-energy-density monochromatic coherent light sources, lasers are widely used in oral medicine. They are extensively used in medicine for soft tissue excision and coagulation (Wenande et al., 2020; Haghsay Khashechi et al., 2024). The combined application of PDT and laser can complement each other and impart synergistic pharmacological effects. Specifically, the thermal effect of lasers can directly solidify and ablate the lesion tissue, and simultaneously modulating mucosal permeability to form delivery microchannels for PSs. The combination of the two can not only aggravate the damage to the deep lesions, but also reduce the risk of thermal damage and scar formation caused by laser treatment (Manimaran et al., 2023). While treating OLK, some researchers performed Er:YAG laser pretreatment of the lesion surface to ablate the keratinized layer and increase the penetration depth of PSs, followed by PDT. They found that the lesion regression rate of the combined treatment group was significantly higher than that of the simple PDT group, and the recurrence rate was lower (Yan et al., 2022). It should be noted, however, that laser pretreatment demands precise control of irradiation parameters—including fluence, pulse duration, and spot size—to ablate the keratinized layer without inflicting unintended thermal injury to the underlying viable tissue. Suboptimal parameter settings may paradoxically increase tissue damage and postoperative discomfort, underscoring the operator-dependent nature of this combined approach (Beer et al., 2012; Shan et al., 2022).

### 4.3 PDT + immunotherapy

PDT not only directly kills tumor cells but also induces ICD, which activates the anti-tumor immune response of the body. But the immunosuppressive tumor microenvironment (TME) often limits the durability of this response. The combination of PDT with immune checkpoint inhibitors or other immunomodulators may have a synergistic pharmacological effect (Shi et al., 2023). In animal models of oral squamous cell carcinoma and precancerous lesions, PDT combined with anti-programmed cell death protein 1 (anti-PD-1) antibody therapy significantly prolonged survival and induced stronger tumor-specific T cell responses and immune memory formation. Moreover, PDT can be combined with immune-enhancing strategies, such as DC-based therapeutic approaches and cytokines (e.g., IL-2, IFN-γ) to further amplify its immune activation effects. A critical clinical consideration is that immune checkpoint inhibitors carry a risk of systemic immune-related adverse events (irAEs), including dermatitis, colitis, hepatitis, and endocrinopathies (Postow et al., 2018; Yin et al., 2023). When PDT-induced immunogenic cell death further amplifies immune activation, the risk and severity of these off-target inflammatory responses may be heightened—a concern that remains underexplored in the context of OMLs.Although clinical studies on the combined application of PDT and immunotherapy in OMLs are at an early stage, the success of this approach in the field of tumor immunotherapy provides an important reference for comprehensively treating oral precancerous lesions and early-stage oral cancer (Li et al., 2024).

## 5 Clinical applications of PDT in oral mucosal lesions

PDT has been investigated in the context of five oral mucosal diseases, including herpes simplex, oral candidiasis, leukoplakia, lichen planus, and oral mucositis. The spectrum of photosensitizers examined is relatively broad, encompassing not only solutions of traditional phenothiazinium dyes (MB, TB), but also 5-ALA prodrugs, chlorins (such as Ce6), naturally derived curcumin, and food-grade colorant PSs. The evidence base spans *in vitro* assays, animal models, and clinical trials. These investigations are summarized in Table 2.

**TABLE 2:** PDT treatment parameters, PSs, and clinical outcomes across oral mucosal diseases.

Disease	Photosensitizer and concentration	Light source/Parameters	Study design	Key clinical outcomes	Study
Oral Herpes Simplex	Fotoditazin	AFS device (Polironic, Russia); chlorin-based PS, λ∼660 nm	*In vitro*	First report: effective inactivation of HSV in culture media	Makarov et al. (2014)
	5-ALA	630 nm (LED/laser)	*In vitro*	Dose-dependent effect: 0.1 mg/mL significantly more effective than 0.05 mg/mL in inactivating HSV-1 and inhibiting intracellular HSV replication	Farzaneh et al. (2024)
	Riboflavin	LED (blue light, riboflavin λmax ∼440–450 nm)	*In vitro*	Suppressed HSV-1 attachment to host cells and inhibited viral replication	Pourhajibagher et al. (2025)
	Methylene Blue (MB)	660 nm diode laser, 40 mW, 120 J/cm^2^, 120 s/point	Clinical	Significantly shortened lesion healing time; markedly reduced recurrence frequency	Lago et al. (2020)
	Methylene Blue (MB)	660 nm diode laser	Clinical	MB-PDT demonstrated direct antiviral effect — interference with viral replication, significantly ↓ local viral load	Ajmal (2021)
	Methylene Blue (MB)	660 nm diode laser	Clinical	MB-PDT exerted immune-modulatory effect — downregulation of IL-6 and TNF-α; synergistic dual-action confirmed when combined with direct antiviral effect	Vellappally et al. (2022)
Oral Candidiasis	Methylene Blue (MB)	660 nm diode laser	*In vitro*	Significant reduction in *Candida* counts; fungicidal efficacy superior to nystatin alone	Adnan and Jawad (2024)
	Photogem (chlorin-based)	LED (455 nm and 630 nm), 305 J/cm^2^	*In vitro* + *in vivo* (immunosuppressed mouse)	*In vitro*: significantly reduced *C. albicans* CFUs; *in vivo*: consecutive 5-day PDT markedly cleared fungal colonization on tongue and ameliorated pathological lesions	Freire et al. (2016)
	Toluidine Blue (TB)	633 nm LED, 200 mW/cm^2^	*In vitro*	Inhibited growth of different clinical isolates; reduced *Candida* adhesion to buccal mucosal epithelial cells	Soares et al. (2009)
​	Toluidine Blue (TB)	660 nm GaAlAs laser, 245 J/cm^2^	*In vivo* (rat dorsal tongue)	Significantly reduced *C. albicans* counts vs. control group (p < 0.05); no damage to normal tissues — confirmed safety	Junqueira et al. (2009)
	Toluidine Blue (TB)	660 nm diode laser 180J/cm^2^	Prospective RCT (denture stomatitis)	Complete *Candida* eradication within short period; clearance efficacy against *C. krusei* significantly superior to conventional antifungal therapy (p < 0.05); faster clinical symptom improvement	Rakasevic et al. (2024)
	Indocyanine green (ICG)	808–810 nm diode laser	Double-blind RCT (denture stomatitis)	ICG-PDT + nystatin: 89.3% clinical improvement vs. nystatin alone: 53.6%; significantly superior *Candida* clearance; no adverse effects	Afroozi et al. (2019)
Oral Leukoplakia	5-ALA	635 nm LED, 100 mW/cm^2^	Clinical	ORR 68% (CR 2%, PR 56%); homogeneous OLK responded significantly better than non-homogeneous OLK (P = 0.041)	Wang et al. (2024)
	5-ALA	635 nm LED, 100 mW/cm^2^	Clinical	CR 84.51%; significant efficacy for mild–moderate dysplasia but unsatisfactory outcomes for severe dysplasia; at 1-year follow-up, recurrence associated with persistent adverse habits	Ou et al. (2022)
	Toluidine Blue (TB)	630 nm LED	Clinical	Overall efficacy 86.7% (CR 40%, PR 46.7%); lesion area significantly reduced post-treatment (p < 0.05); continued lesion reduction from treatment end through 1-year follow-up	Di Stasio et al. (2020)
	5-ALA	635 nm LED, 100 mW/cm^2^	Clinical	8 CR, 16 PR; only 2 recurrences during follow-up period	Chen et al. (2019)
	—	—	Systematic review	PDT considered a useful therapeutic strategy for oral precancerous lesions	Bordoloi et al. (2024)
Oral Lichen Planus	ALA	630 nm diode lamp, 300 mW	Clinical	Mean lesion area ↓ 62.91%; overall improvement 87.9%; 37.09% of lesions achieved complete healing	Sulewska et al. (2025)
	Chlorin e6	Semiconductor laser (Haemato LS PDT 660), 660 nm, ≤300 mW, 90 J/cm^2^	Clinical	Mean lesion reduction 55%; overall remission 81.25%	Sobaniec et al. (2013)
	Methylene Blue (MB)	632 nm laser, 120 J/cm^2^	Clinical	50% of lesions improved; mean area reduction 53.3%	Sadaksharam et al. (2012)
	Phenothiazinium chloride	660 nm Semiconductor laser	Clinical	100% clinical response (CR 35%, PR 65%) — highest reported response rate	Cosgarea et al. (2020)
	Methylene Blue (MB)	632 nm diode laser, 120 J/cm^2^, 2 min/lesion	Clinical	Pain scores ↓ up to 80%; significant control of burning sensation	Aghahosseini et al. (2006)
Oral Mucositis	Photogem (chlorin-based)	LED (630 ± 10 nm), 18 J/cm^2^, 100 mW/cm^2^	*In vivo* (hamster, 5-FU-induced OM)	Significantly reduced OM clinical severity by experimental day 10 (p < 0.05); promoted mucosal keratinization and orderly collagen deposition in lamina propria	Cruz Éde et al. (2015)
	Erythrosine	532 nm LED, 26 J/cm^2^, 138 mW/cm^2^, 188 s	*In vivo* (hamster, 5-FU-induced OM)	Reduced OM clinical severity and total area of ulceration on day 10 (p < 0.05); reduced inflammation and tissue damage histologically	de Cássia Dias Viana Andrade et al. (2022)
	Photogem (chlorin-based)	LED (630 ± 10 nm), 250 mW/cm^2^, 250 s	Clinical RCT (chemoradiotherapy-induced OM)	Significantly lower oral mucositis grade on day 14, 21, 28 (p < 0.05); lesion size reduced significantly with PDT vs. laser only (p < 0.05)	Cruz Éde et al. (2015)
	Methylene Blue (MB)	660 nm diode laser, 100 mW, 0.028 cm^2^ spot, 3 J/point	Randomized single-blind pilot (self-controlled, bilateral)	Significantly improved WHO OTS and NCI-CTC scores on PDT-treated side vs. sham-irradiated contralateral side at all treatment time points (p < 0.05)	Lavaee et al. (2020)
	Curcumin	Blue LED (455 nm), 4.3 J, 1.2 W, 1.2 W/cm^2^	Multicenter RCT (3 arms: nystatin vs. PBM vs. PDT)	PDT group: significantly reduced mucositis severity from day 21; markedly lower pain scores; superior *Candida* clearance vs. both PBM group and nystatin control group	de Cássia Dias Viana Andrade et al. (2022)

### 5.1 Infectious oral mucosal lesions

#### 5.1.1 Oral herpes simplex

Oral herpes simplex is a mucocutaneous disease caused by herpes simplex virus (HSV) and is clinically characterized by clustered vesicles. Although the disease is self-limiting, it is highly prone to recurrence. Nucleoside anti-viral agents are the main treatment drugs for this disease; however, they can only inhibit viral replication and alleviate symptoms, but cannot eliminate the virus latent in nerve ganglia to prevent recurrence. Additionally, their prolonged use may induce the emergence of drug-resistant strains. Therefore, novel therapeutic strategies need to be developed for clinical applications.

As described in Section 3.4, ROS generated during PDT mediate virucidal activity through multi-target physicochemical damage to lipids, proteins, and nucleic acids. In the context of HSV infection, these general oxidative mechanisms exploit pathogen-specific vulnerabilities, rendering HSV particularly susceptible to PDT-mediated inactivation The HSV envelope is enriched in glycoproteins gD and gB, which contain multiple disulfide bridges critical for maintaining their functional conformations. Gly-coprotein gD mediates initial viral attachment to host cell receptors such as herpes vi-rus entry mediator (HVEM) and nectin-1, while gB orchestrates subsequent membrane fusion. ROS-mediated oxidative cleavage of these disulfide bonds induces conforma-tional changes that abolish receptor-binding and fusion capacity, thereby blocking vi-ral entry at multiple steps. This structural vulnerability distinguishes HSV from non-enveloped viruses and explains the particularly high efficacy of PDT against her-pesviruses compared to other viral pathogens (Makarov et al., 2014; Farzaneh et al., 2024). Unlike traditional nucleotide drugs that inhibit a single target of DNA polymerase, the multi-target oxidative damage induced by PDT makes it more difficult for viruses to become drug-resistant through mutations, which provides a unique advantage for managing recurrent herpes. However, the precise molecular mechanism underlying the regulatory effects of PDT on HSV-infected cells, especially its effect on the viral latent-reactivation cycle and the detailed regulatory network of immune response, needs to be further studied.

Multiple studies have shown that PDT is effective against HSV. By conducting *in vitro* studies, Makarov et al. (2014) reported that Fotoditazin-mediated PDT can effectively inactivate HSV in culture media. Farzaneh et al. (2024) reported that PDT mediated by 0.1 mg/mL 5-ALA was significantly more effective than that mediated by 0.05 mg/mL 5-ALA in inactivating HSV-1 and inhibiting the replication of HSV in host cells. Pourhajibagher et al. (2025) demonstrated that riboflavin-mediated PDT can suppress the attachment and replication of HSV-1; the underlying mechanism probably involves the binding of riboflavin to viral surface glycoproteins and viral genomic damage upon photoactivation. Based on these *in vitro* findings, researchers have investigated PDT in clinical settings. Lago et al. (2020) treated oral herpes simplex patients with MB-PDT and found that the healing time was significantly shortened and the recurrence frequency was markedly reduced. Ajmal (2021) and Vellappally et al. (2022) further elucidated the dual mechanism of PDT, where direct anti-viral effects interfere with viral replication and reduce local viral load; moreover, modulation of local immune responses through downregulation of IL-6 and TNF-α results in a synergistic therapeutic effect.

#### 5.1.2 Oral candidiasis

Oral Candidiasis (OC) is caused by fungi such as *C. albicans*. It develops more easily in people with low immune function, such as patients administered chemotherapy, those receiving antibiotics or glucocorticoids for a long time, and the elderly who wear dentures. Traditional treatment mainly relies on antifungal drugs, but long-term use leads to the development of drug resistance, and the effect on *candida* infection in the form of biofilm can result in poor outcomes. Therefore, a new strategy needs to be developed that can both remove fungi and not induce drug resistance.

As described in Section 3.4, ROS generated during PDT exert antimicrobial effects through oxidative damage to cellular structures. In the context of oral candidiasis, these general oxidative mechanisms confront a unique pharmacological challenge posed by *Candida* biofilms—structured microbial communities embedded in a self-produced polysaccharide extracellular matrix that forms on oral mucosal surfaces. This matrix architecture creates a formidable pharmacokinetic barrier to drug penetration, while the metabolically quiescent state of fungi within the biofilm and the presence of highly drug-resistant persister cells render conventional antifungal agents largely ineffective at eradicating established infections. PDT circumvents these resistance mechanisms through a fundamentally different mode of action: ROS-mediated degradation of the extracellular matrix polysaccharides disrupts biofilm structural integrity, enabling photosensitizer penetration into deeper layers where direct oxidative killing of fungal cells occurs, while simultaneously inhibiting hyphal formation to prevent biofilm regeneration (Javed et al., 2014; Sousa et al., 2016; Jordão et al., 2020; Gholami et al., 2022). Because this biofilm disruption operates through non-specific physicochemical chemistry rather than targeting specific fungal metabolic pathways, the likelihood of selecting for resistant strains is substantially reduced compared to conventional antifungal therapy. However, critical mechanistic questions remain unresolved, particularly regarding the specific ROS-mediated degradation pathways for extracellular matrix polysaccharides (e.g., β-glucans, mannans) and the molecular mechanisms by which PDT affects the metabolic state transitions of persister cells from dormancy to active growth, thereby rendering them susceptible to oxidative damage.

Adnan and Jawad (2024) isolated *C. albicans* from 75 patients with oral thrush to prepare fungal suspensions for *in vitro* experiments. Treatment with MB-mediated PDT significantly decreased *Candida* counts; the fungicidal efficacy was higher than that of nystatin alone. Freire et al. (2016) confirmed in an *in vitro* model that PDT can significantly reduce the colony-forming units (CFUs) of *C. albicans*. In an immunosuppressed mouse oral infection model, PDT treatment for five consecutive days effectively eliminated fungal colonies on the tongue and ameliorated pathological lesions. Soares et al. (2009) showed that TB-mediated PDT can inhibit the growth of different clinical isolates and reduce their adhesion to buccal mucosal epithelial cells. In an animal study, Junqueira et al. (2009) found that TB-mediated PDT significantly reduced *C. albicans* counts on the dorsal tongue of rats compared to the control group, without damaging normal tissues; their findings suggest that PDT is a safe and effective antifungal therapeutic modality. Multiple clinical studies have investigated the role of PDT in the treatment of denture stomatitis. Rakašević et al. (2024) conducted a prospective randomized controlled trial (RCT). They found that PDT of denture stomatitis can completely remove *Candida* within a short period, and its removal effect on *Candida krusei* is significantly better than that of traditional antifungal therapy (p < 0.05). Clinical symptoms improve faster, indicating that PDT can overcome antifungal drug resistance. Afroozi et al. (2019) conducted a double-blind RCT and reported that the clinical improvement rate of indole green-mediated PDT combined with mycotomycin in the treatment of denture stomatitis reached 89.3%, significantly higher than that of monomycosin (53.6%), and the removal effect of *Candida* was significantly better. No adverse reactions occurred, which indicated that PDT combined with traditional antifungal drugs can significantly improve the success rate of treatment. These findings broaden the application prospects of PDT in the treatment of oral fungal infections and provide new treatment options for clinicians.

### 5.2 Oral patch and plaque diseases

#### 5.2.1 Oral leukoplakia

OLK is an indelible white plaque or plaque on the oral mucosa that cannot be classified as any other known disease. It is a very common OPMD. OLK is the most prevalent OPMD, with a reported prevalence of approximately 0.2%–4.3% and a malignant transformation rate of approximately 1.1%–40.8%. Traditional methods for treating OLK mainly include drug treatment and local surgical resection. However, certain limitations, such as a significant trauma, high recurrence rates, and functional damage, hinder treatment. Therefore, minimally invasive, targeted, and repeatable strategies need to be developed.

The mechanisms underlying the PDT of OLK primarily involve direct cytotoxicity, vascular targeting, immune modulation, and matrix remodeling. ROS generated by PDT selectively kill abnormally proliferating epithelial cells in OLK by inducing apoptosis, necrosis, and autophagy, while simultaneously damaging neovascularization at the site of the lesion, thereby severing the nutrient supply to abnormal tissue. PDT-induced ICD can activate DCs and T cells, establishing tumor-associated antigen-specific immune responses and immune memory, thereby preventing the recurrence of leukoplakia and malignant transformation. PDT also restores the balance between matrix metalloproteinases (MMPs) and tissue inhibitors of metalloproteinases (TIMPs) by downregulating the expression of MMP-2 and MMP-9 while upregulating TIMPs, thus preserving the integrity of the basement membrane, preventing abnormal cell breakthrough across the basement membrane, and decreasing the risk of malignant transformation (Li et al., 2019; Yang et al., 2021; Le et al., 2022). Compared to conventional surgical excision, PDT has unique advantages, including minimal invasiveness, high selectivity, repeatability, and the ability to activate anti-tumor immunity, providing a novel strategy for treating OLK.

Clinical evidence suggests that 5-ALA is the most commonly used PS for treating OLK. Wang et al. (2024) treated 50 cases of OLK with 20% 5-ALA and reported an overall response rate of 58%, with a complete response (CR) rate of 2% and a partial response (PR) rate of 56%. Homogeneous OLK showed a significantly better response to PDT than non-homogeneous OLK (P = 0.041). In a study involving 60 OLK cases, Ou et al. (2022) achieved a CR rate of 84.51%, with high efficacy for mild-to-moderate dysplasia but unsatisfactory outcomes for severe dysplasia. At the 1-year follow-up, recurrence in some patients was found to be associated with persistent adverse habits. Di Stasio et al. (2020) reported that TB-mediated PDT for OLK had an overall efficacy rate of 86.7%, with a CR rate of 40% and a PR rate of 46.7%. The lesion area decreased significantly after treatment (p < 0.05), with continued reduction from the end of treatment through the 1-year follow-up period, suggesting that TB is a promising PS for PDT. By conducting a systematic review involving 17 studies, Bordoloi et al. (2024) concluded that ALA-PDT is a safe, effective, and well-tolerated treatment for oral premalignant lesions, with clinical outcomes including a reduction in lesion size or complete remission, improvement of symptoms, and a decrease in recurrence. No systemic adverse effects or skin photosensitivity were reported with the topical application of ALA.

#### 5.2.2 Oral lichen planus

OLP is an immune-mediated chronic inflammatory disease of the oral mucosa; it has a prevalence of 0.5%–2.0% and predominantly affects middle-aged women. Based on clinical presentation, OLP can be classified into erosive and non-erosive types. The malignant transformation rate of erosive OLP is approximately 1%–2%, and the WHO has classified it as an OPMD. Conventional treatment of OLP relies primarily on corticosteroids; however, the long-term use of corticosteroids is associated with mucosal atrophy, secondary infections, and systemic adverse effects, along with a high recurrence rate. Therefore, safe and effective alternative treatment strategies need to be developed.

The mechanisms underlying the PDT of OLP are primarily based on its immunomodulatory and anti-inflammatory properties. As described in Section 3.4, ROS induce the apoptosis of infiltrating CD8^+^ T cells in the lesion area, thereby attenuating T cell-mediated autoimmune attack. Simultaneously, PDT decreases the levels of pro-inflammatory cytokines (IL-1β and TNF-α) while increasing the levels of anti-inflammatory cytokines (IL-10 and TGF-β), modulating the balance between Th1 and Th2 and alleviating chronic inflammatory responses. Additionally, PDT suppresses the infiltration of inflammatory cells by inhibiting the NF-κB signaling pathway and decreasing the expression of adhesion molecules. At low doses, ROS can also stimulate the proliferation of keratinocytes and improve microcirculation, thereby promoting erosion healing and tissue repair. Unlike the treatment of oral leukoplakia, PDT for managing OLP aims to suppress excessive immune responses rather than to activate anti-tumor immunity, with the administration of relatively lower treatment doses to avoid excessive damage to normal tissues (Mostafa and Tarakji, 2015; Chintha et al., 2024; Batra et al., 2025; Pietruska et al., 2025).

The therapeutic efficacy of PDT in OLP treatment is supported by multiple clinical studies, with particularly promising application prospects for cases refractory to conventional corticosteroid therapy or those with treatment contraindications. Sulewska et al. (2019) reported that after 5% 5-ALA-PDT treatment, the mean lesion area decreased by 62.91%, with an overall improvement rate of 87.9%, of which 37.09% of lesions healed completely. Sadaksharam et al. (2012) conducted a study using 5% MB-mediated PDT for OLP treatment. They found that 50% of lesions showed improvement, with a 53.3% reduction in the area. RCTs have provided stronger evidence for PDT in the management of OLP. Wang et al. (2026) conducted a single-blinded RCT involving 74 erosive OLP patients and found that 0.1 mg/mL MB-PDT combined with topical corticosteroid achieved a significantly higher clinical efficacy rate than corticosteroid alone (P = 0.006), with sustained improvement in pain through week 12 (P < 0.01) and a significantly lower recurrence rate, with no reported adverse events. Pietruska et al. (2025) performed a randomized clinical trial involving 90 OLP patients and found that 5% ALA-PDT achieved significantly greater and more durable improvements in lesion area, clinical severity, and pain scores compared to topical corticosteroid, particularly on non-keratinized mucosa (mean lesion reduction from 2.64 cm^2^ to 0.56 cm^2^ at 6 months; P < 0.001), whereas the corticosteroid group exhibited relapse at 6 months. These findings collectively confirm that PDT can effectively promote the regression of OLP lesions and healing to different degrees.

### 5.3 Oral ulcerative diseases

#### 5.3.1 Oral mucositis

Oral mucositis (OM) is an inflammatory lesion of the oral mucosa directly induced by the cytotoxic effects of chemotherapy and/or radiotherapy; OM is one of the most common adverse reactions in cancer treatment. OM lesions are frequently colonized by microorganisms such as *C. albicans* and HSV, which further compromise patient prognosis. Conventional treatment approaches primarily consist of pharmacotherapy and physical therapy; however, these modalities generally provide only temporary symptom relief or are accompanied by significant adverse effects. Hence, the prevention and treatment of OM need to be further investigated. In recent years, PDT has become a promising strategy for managing OM.

PDT may exert its therapeutic effects on OM through the following mechanisms: (1) Stimulation of cell metabolism: low-dose light irradiation can promote ATP synthesis, improve mitochondrial function, and accelerate the repair of damaged cells; (2) Anti-inflammatory effects: low-dose PDT (LD-PDT) can downregulate the expression of pro-inflammatory cytokines (IL-1β, IL-6, and TNF-α) while simultaneously increasing the release of anti-inflammatory factors (IL-10 and TGF-β); (3) Promotion of tissue repair: by stimulating the proliferation of fibroblasts, accelerating epithelial regeneration, and promoting angiogenesis, PDT significantly shortens mucosal healing time; (4) Analgesic effects: LD-PDT can modulate the release of pain mediators and reduce neural sensitization (Zadik et al., 2019).

Animal experiments have validated the therapeutic efficacy of PDT. Using a 5-fluorouracil-induced hamster OM model, de Paula et al. (Cruz Éde et al., 2015), found that compared to the untreated group, the PDT group exhibited significantly lower clinical severity of OM by day 10 of the experiment (p < 0.05) and promoted mucosal keratinization and orderly collagen deposition within the lamina propria. The results of a biochemical analysis revealed that PDT can mitigate oxidative stress damage by regulating the activity of superoxide dismutase and catalase without interfering with normal tissue repair processes. Researchers have obtained substantial medical evidence regarding the prevention and treatment of OM. In the prevention and management of radiotherapy-induced OM, the use of PDT has progressively increased in clinical settings. Lavaee et al. (2020) conducted a randomized single-blind clinical pilot study involving 15 patients with chemotherapy-induced bilateral OM. They adopted a self-controlled design in which the intervention side received MB-mediated PDT while the contralateral side received sham laser irradiation only. The results revealed that compared to the control side, the PDT intervention side showed significantly better World Health Organization Oral Toxicity Scale and National Cancer Institute Common Toxicity Criteria scores at all treatment time points (p < 0.05). de Cássia Dias Viana Andrade et al. (2022) conducted a multicenter randomized clinical study involving 30 patients with chemoradiotherapy-related OM. They compared nystatin (control group), low-level laser therapy (photobiomodulation [PBM] group), and curcumin-mediated PDT. They found that the PDT group exhibited significantly lower severity of mucositis from day 21, considerably lower pain scores, and more effective *Candida* clearance compared to the PBM and control groups, confirming that curcumin-mediated PDT is highly effective as an adjunctive treatment for chemoradiotherapy-related OM.

### 5.4 Comparison of clinical efficacy and appraisal of current evidence

The aforementioned clinical studies collectively revealed that PDT demonstrates therapeutic potential across different types of OMLs, with efficacy varying based on the nature of the disease. In antimicrobial applications, PDT yields the strongest evidence for oral candidiasis, achieving clinical improvement rates of 89.3% when combined with antifungal agents (Afroozi et al., 2019), and a better ability to eradicate resistant strains such as *C. krusei* (Rakasevic et al., 2024). For oral herpes simplex, PDT can shorten healing time and reduce recurrence frequency through multi-target oxidative damage, with a low propensity to induce drug resistance (Lago et al., 2020; Ajmal, 2021; Vellappally et al., 2022). When PDT is applied to manage OPMDs, it achieves an overall response rate of 68.0%–86.7% for OLK (Di Stasio et al., 2020; Wang et al., 2024), with favorable outcomes in homogeneous lesions and mild-to-moderate dysplasia, whereas it has suboptimal efficacy against severe dysplasia and non-homogeneous leukoplakia (Ou et al., 2022; Wang et al., 2024). For immune-mediated OLP, PDT exerts its effects primarily through anti-inflammatory and immunomodulatory mechanisms, achieving a 55%–62.91% reduction in lesion area (Sobaniec et al., 2013; Sulewska et al., 2019), and is particularly suitable for refractory cases unresponsive to corticosteroid therapy. Regarding the prophylaxis and management of OM, preliminary evidence suggests that PDT can decrease the severity of mucositis and promote tissue repair (Lavaee et al., 2020; de Cássia Dias Viana Andrade et al., 2022), although high-quality clinical trials remain limited.

With respect to the selection of PSs, MB and TB are the most widely used in clinical practice because they are inexpensive, easy to use, and have a wide range of applications. The compound 5-ALA has an inherent target advantage in the treatment of OLK, because it can be converted to PpIX in active proliferating epithelial cells. Emerging PSs, including ICG and curcumin, have shown promising results in the treatment of oral candidiasis and oral mucositis, respectively.

However, the existing evidence has certain limitations. First, most studies have a small sample size (15–90 patients), insufficient statistical power, and a significant lack of large-scale multi-center RCTs. Second, the concentration of PSs, irradiation wavelength, energy density, and treatment frequency differ significantly, which prevents researchers from directly comparing different studies. Third, the follow-up period usually does not exceed 1 year; therefore, the evaluation of long-term efficacy and safety is not possible. Fourth, the heterogeneity of outcome evaluation criteria highlights that a standardized evaluation framework is needed. These limitations indicate that, although therapeutic prospects are promising, transitioning PDT from clinical exploration to standardized treatment protocols for OMLs is quite challenging.

## 6 Conclusion and prospects

PDT can effectively treat oral malignant tumors through coordinated multi-target pharmacological actions, including direct cytotoxicity, vascular injury, and immune regulation. PDT exhibits high antimicrobial efficacy against OC and herpes simplex with a high genetic barrier to resistance, achieves favorable clinical response rates in OLK, effectively alleviates symptoms, promotes the regression of lesions in OLP, and shows potential in the prophylactic management of OM.

PDT causes minimal collateral damage to normal mucosa since PSs are concentrated in diseased cells, and light is directed to the lesion, not beyond it. If the PSs are applied topically, then systemic uptake are very small, and repeated sessions do not add cumulative toxicity. Photoxicity is the most well-known adverse effect of PDT. Systemic agents can cause patients to become photosensitive for a week, but topical preparations localize this hazard. Pain, erythema, and superficial erosion occur during or shortly after irradiation, but these conditions do not require intervention, and their intensity tracking is done by PSs concentration and light dose. Most published works have a follow-up period of 6–24 months, and the recurrence within 1–2 years affects a significant minority of OLK patients. The question remains whether serial PDT can maintain remission in OLP. Whether the PDT-generated ROS can drive malignant transformation in OPMD tissue that is already genomically unstable is an open question. No reports have been made of excess malignancy, but existing follow-up data are too limited to rule out the risk.

However, standardized clinical applications of PDT still have certain limitations. Besides the heterogeneity of the aforementioned treatment parameters and insufficient high-quality clinical evidence, the disease-specific molecular mechanism that regulates PDT in different oral malignant tumors remains poorly understood. The molecular basis of the difference in treatment response and the intrinsic mechanism underlying drug resistance need to be determined; lack of information regarding these restricts the shift from empirical treatment choice to a precise guidance treatment plan.

Published protocols diverge at nearly every controllable variable. Starting with the PSs, the evidence for dose-dependence is absent. 5-ALA, the most extensively studied prodrug in this field, has been applied at concentrations spanning four orders of magnitude: sub-milligram-per-milliliter solutions *in vitro*, 10% emulsions, and 20% topical preparations in clinical OLK series. Yet no systematic concentration–response study rationalizes any of these choices. MB presents a different deficit. Most clinical reports simply omit the concentration, and among the minority that disclose it, formulations range from 5% aqueous rinses to unspecified gels, precluding any assessment of dose–response relationships. Even within a single indication, the same PS can be applied at concentrations that differ by an order of magnitude: toluidine blue at 0.1 mg/mL versus 1 mg/mL for oral candidiasis, then at 2.5% for OLK, all without published justification. Concentration is only the first variable. Photogem, consistently applied at 5 mg/mL in oral mucositis studies, is paired with energy densities that differ by more than an order of magnitude across reports. Treatment schedules are similarly unanchored. Protocols encompass four sessions within 2 weeks, eight sessions over 1 to 2 months, ten sessions at biweekly intervals, and five-consecutive-day regimens, without a single formal dose-finding or schedule-optimization component in the published literature. Among irradiation parameters, wavelength is the least variable (630–660 nm, reflecting the absorption maxima of the most-used PSs), but energy density spans 1.5 J/cm^2^ to above 300 J/cm^2^, a >200-fold range. Power density varies from 10 mW/cm^2^ to 200–300 mW/cm^2^. Light sources include LEDs, diode lasers, semiconductor lasers, argon-pumped dye lasers, and Xenon arc lamps, each with distinct emission spectra, beam profiles, and tissue penetration characteristics. ALA-PDT for OLK represents the only indication for which treatment guidelines have been proposed (20% ALA, 630 ± 5 nm, 100 mW/cm^2^, 100 J/cm^2^, every 2–3 weeks). Even within this narrow domain real-world protocols deviate considerably, and the guideline authors explicitly note that consensus parameters must await multicenter randomized evidence (Chen et al., 2019).

Future studies should focus on three key directions. At the mechanistic level, multi-omics technologies should be used to systematically elucidate the regulatory effects of PDT on key signaling pathways (NF-κB, MAPK, and JAK-STAT) and immunomodulatory networks, facilitating the discovery of novel biomarkers and therapeutic targets. At the level of development of PSs, next-generation intelligent PSs, particularly stimuli-responsive and near-infrared-II (NIR-II) PSs, are expected to improve targeting specificity and tissue penetration capacity. At the clinical translation level, large-scale multicenter RCTs are needed to establish international standardized guidelines encompassing indication selection, treatment parameters, and efficacy evaluation criteria, while expanding the application of PDT to refractory conditions, such as oral submucous fibrosis and recurrent aphthous ulcers.

To summarize, with a better understanding of the molecular mechanism, the continuous research and development of new PSs, and the establishment of standardized schemes, PDT may become an indispensable therapeutic modality for OMLs, providing patients with safer, more effective, and more individualized treatment options.
